# The impact of administration of tranexamic acid in reducing the use of red blood cells and other blood products in cardiac surgery

**DOI:** 10.1186/1471-2253-6-9

**Published:** 2006-08-30

**Authors:** Alain Vuylsteke, Palanikumar Saravanan, Caroline Gerrard, Fay Cafferty

**Affiliations:** 1 Department of Anaesthesia, Papworth Hospital NHS Foundation Trust, Papworth Everard, Cambridgeshire, CB3 8RE, UK; 2 Research and Development Department, Papworth Hospital NHS Foundation Trust, Papworth Everard, Cambridgeshire, CB3 8RE, UK

## Abstract

**Background:**

To study the effect of administration of tranexamic acid on the use of blood and blood products, return to theatre for post-operative bleeding and the length of intensive care stay after primary cardiac surgery, data for 4191 patients, of all priorities, who underwent primary cardiac operation during the period between 30/10/00 and 21/09/04 were analysed.

**Methods:**

Retrospective analysis of data collected prospectively during the study period. The main outcome measures were whether or not patients were transfused with red blood cells, fresh frozen plasma or any blood product, the proportion of patients returned to theatre for investigation for post-operative bleeding and length of stay in the intensive care unit. We performed univariate analysis to identify the factors influencing the outcome measures and multivariate analysis to identify the effect of administration of tranexamic acid on the outcome measures.

**Results:**

Administration of tranexamic acid was an independent factor affecting the transfusion of red blood cells, fresh frozen plasma or any blood product. It was also an independent factor influencing the rate of return to theatre for exploration of bleeding. The odds of receiving a transfusion or returning to theatre for bleeding were significantly lower in patients receiving tranexamic acid. The administration of tranexamic acid also significantly decreased blood loss. We did not find any association between the administration of tranexamic acid and the length of intensive care stay.

**Conclusion:**

Based on the analysis of 4191 patients who underwent a primary cardiac operation, administration of tranexamic acid decreased the number of patients exposed to a transfusion or returned to theatre for bleeding in our institute.

## Background

Tranexamic acid is an antifibrinolytic agent used in the control of bleeding under various circumstances [1] and has been used to minimise bleeding after cardiac surgery [2,3]. In cardiac surgery, postoperative bleeding is associated with an increased incidence of surgical re-exploration to identify the source of bleeding; increased use of blood; significant morbidity such as renal failure, sepsis, arrhythmias, prolonged requirement for mechanical ventilatory support and longer hospital stay; and increased mortality [4-8].

Randomised prospective studies have shown that tranexamic acid can decrease exposure to blood transfusions [9-14]. Unfortunately, those studies used a variety of dose regimens and gave diverse conclusions, making it difficult to translate these findings to the clinical arena with confidence that it will indeed be a useful treatment. However, our transfusion hospital guidelines have stated for the last few years that we should administer tranexamic acid at a dose of 2 g intravenously for any cardiac operation that involves use of cardiopulmonary bypass and where aprotinin is not administered.

In an effort to control the cost of transfusion and to ensure that all blood products are only used where appropriate, our hospital employs a clinical information analyst who is solely responsible for collecting prospectively, continuously and at the bedside, all data related to transfusions in connection with cardiac surgery. Compliance with guidelines is constantly assessed and reported and data reviewed by this analyst showed that the institutional antifibrinolytic guidelines were, in fact, not strictly adhered to (Table 1).

**Table 1:** Variation in tranexamic acid administration between anaesthetists at this hospital, within the timeframe studied.

	Number of patients (%)		
	
Anaesthetist	Total	Tranexamic acid	No Tranexamic acid
1	624 (14)	519 (83)	105 (17)
2	453 (11)	255 (56)	198 (44)
3	437 (10)	385 (88)	52 (12)
4	437 (10)	346 (79)	91 (21)
5	421 (10)	380 (90)	41 (10)
6	344 (8.2)	282 (82)	62 (18)
7	342 (8.2)	293 (86)	49 (14)
Other	1133 (27)	899 (79)	234 (21)

Variation in tranexamic acid administration, between anaesthetists at this hospital, for individual anaesthetists performing more than 300 cases within the timeframe studied. Other anaesthetist includes all the anaesthetists who each performed less than 300 cases in the timeframe studied. The proportion of patients receiving tranexamic acid within this group varied from 56% to 90% depending upon anaesthetist.

We therefore decided to investigate the impact of tranexamic acid administration on the use of red blood cells and other blood products using the data collected prospectively in our hospital from 30/10/00 to 21/09/04. This reports comes at a time when the safety of aprotinin is reviewed in light of recent evidence suggesting that aprotinin may have a major negative impact on outcome and that alternatives drugs, such as tranexamic acid, may not only be cheaper but also safer alternatives [15,16]. Aprotinin is an antifibrinolytic agent whose efficacy on decreasing perioperative transfusion has been extensively and rigorously documented [17] and concerns on its safety has indeed prompted numerous reactions [17-20].

## Methods

To answer our specific question, we performed a retrospective analysis on prospectively collected data. The data was collected between 30/10/00 and 21/09/04 in Papworth Hospital, Cambridge, UK.

### Data collection

Our hospital employs a full time clinical information analyst who is solely responsible for continuously collecting all data relevant to blood loss and blood transfusions from all patients undergoing cardiac surgery. The data collection is prospective and occurs daily at the bedside as part of the ongoing and continuous audit process in the hospital. These data are audited regularly and information fed back to all staff to ensure effective use of blood and blood products. Initially these audits were conducted for a specified number of weeks, but now the audit process is continuous. Each audit now runs from 1^st ^January to 31^st ^December, for the purposes of analysis.

### Transfusion guidelines

**Figure 1:**
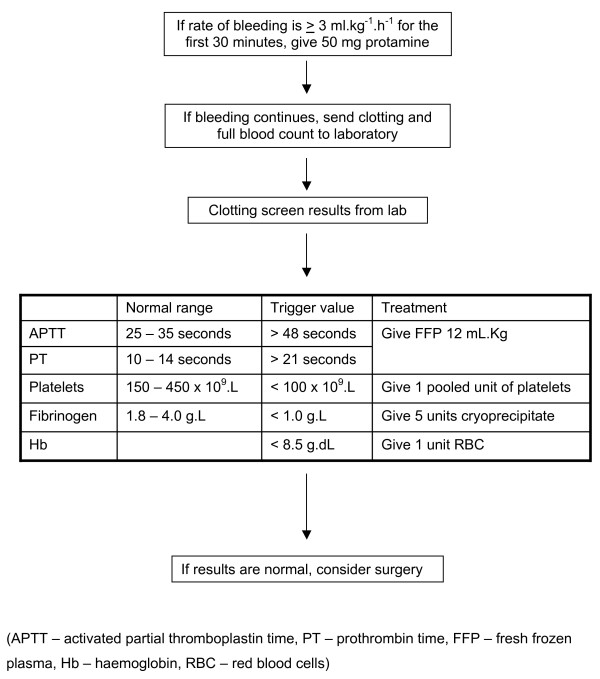
Guidelines for the administration of blood products at our institution.

**Figure 2:**
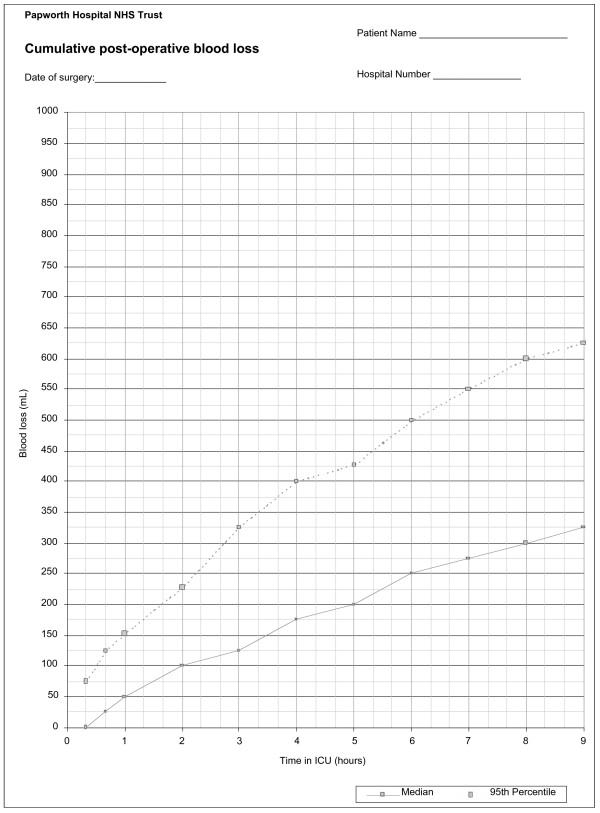
Record of cumulative blood loss, used prospectively to assess post-operative blood loss. Median and 95^th ^centile values are based on post-operative blood loss from first time coronary artery bypass graft patients who did receive any blood products and who were not returned to theatre for bleeding.

Specific guidelines exist for the use of all blood products throughout the hospital (Figure 1). These guidelines were based on data collected from a previous audit, literature review and institutional consensus. To support medical decision, we routinely use a graph to represent the cumulative post-operative blood loss over time in our patients against a representation of the median blood loss of untransfused patients not returned to theatre for excessive bleeding (Figure 2). Adherence to the guidelines is monitored daily by the clinical information analyst.

### Antifibrinolytic administration

We have an institutional protocol for administration of antifibrinolytic agents stating that tranexamic acid should be given to all patients undergoing cardiopulmonary bypass and not receiving aprotinin. Whenever tranexamic acid is administered, it is given as an intravenous injection of 2 g in total with 1 g before and 1 g after cardiopulmonary bypass.

### Inclusion and exclusion criteria

The data from all patients, of all priorities, who underwent primary coronary artery bypass graft (CABG) surgery, single valve surgery (either repair or replacement) or combined CABG and single valve operations during the period 30/10/00 to 21/09/04 were included for the analysis. Patients who received aprotinin were excluded.

### Outcomes of interest

Blood loss and blood transfusion are common occurrences after cardiac surgery and are associated with surgical re-exploration. Our aim was to identify whether administration of tranexamic acid, in our clinical setting, had an effect on blood transfusion and if its administration was an independent factor influencing whether a patient would be taken back to theatre for re-exploration or stay longer in the intensive care unit (ICU). We considered these outcomes as a binary measure and selected five primary outcomes of interest:

a. Whether or not a patient received a transfusion of red blood cells (RBC)

b. Whether or not a patient received a transfusion of fresh frozen plasma (FFP)

c. Whether or not a patient received any transfusions (including RBC, FFP, platelets and cryoprecipitate)

d. Whether or not a patient had to return for surgical re-exploration

e. Whether or not a patient stayed in ICU for more than 1 day.

Another outcome measure was added on request of the Journal Editor, asking whether the administration of tranexamic acid decreased blood loss.

### Analysis of the data

The analysis of the data was done in four steps:

(1) Initially, we compared the baseline characteristics of the patients who received tranexamic acid (TA group) and who did not receive tranexamic acid (NTA group). These were age, sex, body mass index (BMI), priority of the surgery (elective, urgent or emergency), type of surgery (CABG, valve or combined), additive EuroSCORE. As the data collection was part of the audit process, we also assessed whether there was difference in the proportion of patients receiving tranexamic acid over time, by comparing the number of patients treated in each audit. Tests used were Chi-square test (categorical variables), Mann-Whitney U test and two-sample Student t-test (continuous variables).

(2) In the second step, we used univariate logistic regression to assess the effect of administration of tranexamic acid on each of the 5 main outcomes of interest. We also used this method to identify other covariates that had an effect on the outcome measures. The covariates considered were the baseline characteristics as listed above, as well as time of audit, preoperative haemoglobin, bypass time, preoperative aspirin use, preoperative clopidogrel use, the anaesthetist involved and the surgeon. Preoperative aspirin and clopidogrel use were analysed without taking into consideration the time of administration of the drug. Those patients recorded as having taken the drug, irrespective of the time when they were stopped, were considered as using the drug preoperatively. The covariates surgeon and anaesthetist were analysed using one surgeon and one anaesthetist as a reference respectively and assessing the effects of others by univariate analysis.

(3) In the third step, we built up a multiple logistic regression model (initially using forward selection and then verified by backward elimination) to adjust for the effects of the baseline characteristics and the other covariates and to assess whether the administration of tranexamic acid had an independent effect on the outcome measures.

(4) In the fourth step, we performed two subgroup analyses of the data.

(4.1) The first analysis considered only patients who received a transfusion. Our analysis so far had looked at transfusion as a binary outcome; that is, whether or not patients received a transfusion. Here, we used a Mann-Whitney U test to assess whether there was any difference in the amount of red blood cells and other blood products transfused in the two groups (TA and NTA).

(4.2) The second analysis considered only patients who did not return to theatre for investigation of excessive blood loss. Patients who returned to theatre belong to a high-risk group for receiving transfusions and are likely to stay longer in ICU. It was a concern, therefore, that these patients may obscure trends in the data relating to tranexamic acid. Hence, we excluded the patients who returned to theatre for investigation of excessive blood loss and repeated the same statistical tests on this new group to identify the effects of administration of tranexamic acid on the outcome measures of interest.

(5) In the final step we considered the blood loss outcome. Total blood loss and 12 hour blood loss were compared between the TA and NTA groups using the Mann-Whitney U test. Separate comparisons were made for those who were and were not returned to theatre for further investigation. Multivariate linear regression was then performed to assess which covariates had an independent effect on total blood loss. The same set of covariates was considered, and stepwise procedures were performed, as before. Since the distribution of blood loss was positively skewed, the outcome was transformed prior to modelling using the natural log transformation. Results are therefore reported as the proportionate change in blood loss attributable to a given covariate (i.e. the exponentiated coefficient corresponding to that covariate).

Statistical analyses were performed using SPSS (Statistical Package for the Social Sciences) for Windows, version 12.0.

This work has not required ethical approval as it is based on information collected as part of a routine audit program, and Local Caldecott Guardian approval was obtained for publication of these data.

## Results

We identified data for 4191 patients who had undergone primary coronary artery bypass grafting surgery (CABG), single valve surgery (repair or replacement) or both procedures combined between 30/10/00 and 21/09/04. Baseline characteristics of patients are shown in Table 2. Seventy five percent (n = 3153) of the patients were men and 70 percent of all patients (n = 2933) were scheduled for CABG. Forty seven percent of all patients (n = 1970) received a transfusion and 5.5 percent of all patients (n = 229) returned to theatre for investigation of excessive blood loss.

**Table 2:** Baseline characteristics of the patient cohort analysed.

**Variable **	**Whole Cohort **	**TA group **	**NTA group **	**p value** **TA vs. NTA **
All patients (n, %)	4191	3359 (80)	832 (20)	-
Sex (n, %)				
Male	3153 (75)	2537 (76)	616 (74)	0.373
Priority (n, %)				
Elective	3437 (82)	2750 (82)	687 (82)	0.839
Urgent	687 (16)	556 (16)	131 (16)	
Emergency	67 (2)	53 (2)	14 (2)	
Surgery type (n, %)				
CABG	2933 (70)	2338 (70)	595 (72)	0.035
Valve	818 (20)	648 (19)	170 (20)	
Combined	440 (10)	373 (11)	67 (8)	
Mean Age (SD)	67 (10.2)	67.2 (10.2)	66.3 (10.3)	0.019
Mean BMI (SD)	27.5 (4.4)	27.5 (4.4)	27.7 (4.6)	0.215
EuroSCORE (Median, IQR)	4 (2,6)	4 (2,6)	4 (2,6)	0.014
Transfusion (n, %)				
RBC	1933 (46)	1513 (45)	420 (50)	0.005
FFP	383 (9.1)	288 (8.6)	95 (11)	0.011
Any blood products	1970 (47)	1543 (46)	427 (51)	0.005
Return to theatre (n, %)	229 (5.5)	160 (4.8)	69 (8.3)	< 0.001
ICU stay > 1 day (n, %)	933 (22)	735 (22)	198 (24)	0.234

Baseline characteristics of the patient cohort analysed. Patients who were in the tranexamic acid group were older, had a higher EuroSCORE (mean (SD) 4.61 (3.12) compared to4.36 (3.13) in the NTA group) and higher proportion of them underwent combined procedures. Despite this, the rates of transfusion of RBC, FFP, all blood products and the rate of return to theatre for bleeding were lower for patients in this group. (TA – tranexamic acid, NTA – no tranexamic acid, SD – Standard Deviation, CABG – coronary artery bypass grafting, Valve – single valve repair or replacement, combined – coronary artery bypass grafting and single valve surgery, BMI – body mass index, RBC – red blood cells, FFP – fresh frozen plasma, ICU – intensive care unit, n – number, IQR – inter quartile range)

Eighty percent of all patients (n = 3359) received tranexamic acid (TA group). There were no differences between the groups with respect to sex, priority of surgery and BMI. When compared to the overall baseline characteristics, patients in the TA group were older, had a higher EuroSCORE and had a higher proportion of combined operations. The differences in age and EuroSCORE, though statistically significant were not clinically significant (Table 2). It was noted that the proportion of patients receiving tranexamic acid has increased over the last four years (Table 3). The difference in the proportion of patients receiving tranexamic acid over time was found to be statistically significant (p < 0.001, Chi-Square test).

**Table 3:** Trend in the administration of tranexamic acid over time.

		Number of patients (n = 4191)		
		
Audit	Time frame	Total (%)	TA group (%)	NTA group (%)
1	30/10/00 – 18/02/01	348 (8)	247 (71)	101 (29)
2	23/04/01 – 02/09/01	413 (10)	277 (67)	136 (33)
3	12/11/01 – 20/12/02	1345 (32)	1139 (85)	206 (15)
4	20/01/03 – 31/12/03	1136 (27)	951 (84)	185 (16)
5	01/01/04 – 21/09/04	949(23)	745 (78)	204 (22)

Trend in the administration of tranexamic acid over time. Implementation of new antifibrinolytic guidelines resulted in an overall increase in the number of patients receiving tranexamic acid (p < 0.001, Chi-Square test, significant). (TA – tranexamic acid, NTA – no tranexamic acid)

A higher proportion of patients in the no tranexamic acid (NTA) group received a transfusion and returned to theatre for investigation of excessive blood loss. Univariate logistic regression analysis showed that the odds of receiving a transfusion of red blood cells, fresh frozen plasma or any blood product or returning to theatre for excessive blood loss were lower in TA group (Table 4). However, we did not find any difference in the proportion of patients remaining in ICU beyond one day between the groups.

**Table 4:** Univariate analysis of the effects of tranexamic acid administration on outcomes of interest.

Outcome measure	TA group (n = 3359)	NTA group (n = 832)	Odds ratio (95% CI)	p value
RBC transfusion (n, %)	1513 (45)	420 (51)	0.80 (0.69, 0.94)	0.005
FFP Transfusion (n, %)	288 (8.6)	95 (11)	0.78 (0.57, 0.93)	0.011
Any blood product transfusion (n, %)	1543 (46)	427 (51)	0.81 (0.69, 0.94)	0.005
Return to Theatre (n, %)	160 (4.8)	69 (8.3)	0.55 (0.41, 0.74)	<0.001
ICU stay > 1 day (n, %)	735 (22)	198 (24)	0.90 (0.75, 1.07)	0.234

Univariate analysis of the effects of tranexamic acid administration on outcomes of interest. The univariate analysis showed that the odds of receiving a transfusion or returning to theatre for bleeding were less in patients in tranexamic acid group, but that there was no difference in the proportion of patients staying for more than 1 day

(TA – tranexamic acid, NTA – no tranexamic acid, RBC – red blood cells, FFP – fresh frozen plasma, ICU – intensive care unit, CI – Confidence interval, n – number) (p < 0.05, statistically significant)

Univariate logistic regression was also used to identify if other covariates had an effect on the outcome measures. The results of this univariate analysis are given in Table 5. All the covariates were found to affect at least one of the outcome measures. Patients were more likely to receive a transfusion if they were female, were older, had an emergency operation, had a combined procedure, had a lower BMI, had a higher EuroSCORE, had lower haemoglobin preoperatively, used clopidogrel preoperatively or did not use aspirin preoperatively when compared to the baseline characteristics of the group. The surgeon and the anaesthetist also influenced on whether or not patients received a transfusion and the overall effect was significant (Table 6, overall p value < 0.05).

**Table 5:** Univariate analysis of the other covariates.Univariate analysis of the other covariates.

	**Odds Ratio (95% Confidence Interval) **				
	
Covariates	RBC transfusion	FFP transfusion	Any blood product transfusion	Return to theatre	ICU stay
Male	Reference^1^	Reference^1^	Reference^1^	Reference^1^	Reference^1^
Female	4.95 (4.23, 5.79)*	1.29 (1.02, 1.62)*	4.83 (4.13, 5.65)*	0.77 (0.56, 1.08)*	1.38 (1.17, 1.62)*
Elective	Reference^1^	Reference^1^	Reference^1^	Reference^1^	Reference^1^
Urgent	2.83 (2.38, 3.36)*	2.45 (1.93, 3.12)*	2.98 (2.50, 3.54)*	1.79 (1.31, 2.45)*	2.68 (2.25, 3.20)*
Emergency	9.18 (4.53, 8.58)*	7.65 (4.59, 12.77)*	10.20 (4.86, 21.42)*	2.31 (1.04, 5.14)*	13.21 (7.57, 23.05)*
CABG	Reference^1^	Reference^1^	Reference^1^	Reference^1^	Reference^1^
Valve repair	0.87 (0.66, 1.16)	1.84 (1.16, 2.90)*	0.95 (0.72, 1.26)	0.65 (0.28, 1.50)	0.95 (0.67, 1.34)
Valve replacement	1.56 (1.31, 1.86)*	2.40 (1.81, 3.17)*	1.60 (1.34, 1.91)*	1.78 (1.24, 2.55)*	0.90 (0.72, 1.13)
CABG + Valve	4.02 (3.21, 5.03)*	4.76 (3.64, 6.22)*	3.94 (3.15, 4.94)*	3.50 (2.51, 4.87)*	2.45 (1.98, 3.02)*
Age	1.07 (1.06, 1.08)*	1.05 (1.04, 1.07)*	1.07 (1.06, 1.07)*	1.05 (1.03, 1.06)*	1.04 (1.03, 1.04)*
BMI	0.93 (0.92, 0.94)*	0.88 (0.86, 0.91)*	0.93 (0.92, 0.94)*	0.94 (0.91, 0.97)*	1.01 (0.99, 1.03)
EuroSCORE	1.37 (1.32, 1.41)*	1.23 (1.19, 1.27)*	1.38 (1.34, 1.41)*	1.12 (1.09, 1.16)*	1.25 (1.22, 1.28)*
Hb	0.50 (0.47, 0.53)*	0.90 (0.84, 0.96)*	0.56 (0.53, 0.58)*	1.00 (0.98, 1.03)	0.78 (0.74, 0.81)*
Bypass time	1.02 (1.01, 1.02)*	1.02 (1.02, 1.03)*	1.02 (1.01, 1.02)*	1.01 (1.01, 1.02)*	1.01 (1.01, 1.01)*
Aspirin use	0.83 (0.72, 0.96)*	0.58 (0.46, 0.72)*	0.80 (0.69, 0.92)*	0.98 (0.72, 1.34)	1.07 (0.90, 1.27)
Clopidogrel use	1.58 (1.31, 1.91)*	1.58 (1.18, 2.10)*	1.58 (1.30, 1.91)*	1.40 (0.97, 2.04)	1.88 (1.53, 2.31)*

The covariates with nominal data were analysed with one of their category as reference (^1^) and the others compared to the reference. The univariate analysis of the other covariates showed their influence on the outcome measures studied, independent of tranexamic acid usage. (* = p < 0.05, statistically significant) (RBC – red blood cells, FFP – fresh frozen plasma, ICU – intensive care unit, BMI – body mass index, Hb – preoperative haemoglobin).

**Table 6:** Effect of Surgeon and Anaesthetist on outcomes of interest.

	Surgeon		Anaesthetist	
	
Variable	Range (as %)	Overall p value	Range (as %)	Overall p value
TA administration	--	--	56 – 90	<0.001
RBC transfusion	41 – 51	0.007	41 – 52	0.028
FFP transfusion	5 – 15	<0.001	7 – 11	0.573
Any blood products transfusion	41 – 53	0.002	42 – 53	0.019
Return to theatre	3.6 – 8.1	0.011	3.5 – 7.6	0.146
ICU stay (> one day)	17 – 33	<0.001	17 – 27	0.005

Effect of Surgeon and Anaesthetist on outcomes of interest. The surgeon had an influence on the rate of transfusion of red blood cells, fresh frozen plasma and all blood products, the rate of return to theatre for bleeding and the rate of intensive care stay longer than one day. The anaesthetist had an influence on the rate of tranexamic acid administration, the rate of transfusion of red blood cells and all blood products and the rate of intensive care stay longer than one day. (TA – Tranexamic acid, RBC – red blood cells, FFP – fresh frozen plasma, ICU – intensive care unit, Range – proportion of patients among various surgeons and anaesthetists) (* = p value < 0.05, statistically significant)

This analysis also showed that the odds of being returned to theatre were higher if the patients were male, were older, had a lower BMI, had a higher EuroSCORE, had a longer bypass time, had an emergency operation or had a combined operation (Table 5). The surgeon, but not the anaesthetist, also had an influence on the likelihood of a patient returning to theatre (Table 6, overall p value for surgeon < 0.05). However, preoperative haemoglobin and use of aspirin or clopidogrel did not show any association with the rates of return to theatre. Patients were more likely to stay for greater than 1 day in the ICU if they were older, were female, had a higher EuroSCORE, had lower preoperative haemoglobin, used clopidogrel preoperatively, had an emergency operation or had a combined operation when compared to the baseline characteristics. BMI did not have an effect on the length of ICU stay when assessed as a continuous variable.

After consideration of all of the above covariates, the final multivariate model for predicting red blood cell transfusion incorporated terms for audit time, age, BMI, EuroSCORE, sex, priority, surgery type, preoperative haemoglobin, bypass time and surgeon. Aspirin use, clopidogrel use and anaesthetist were found to be non-significant after adjustment for other covariates. The multivariate model for predicting any blood product transfusion incorporated the same terms as the red blood cell model, as did the FFP model, with the exception of sex and haemoglobin, which were found to be non-significant after adjustment for other covariates. The model predicting returns to theatre incorporated all of the above terms with the exception of haemoglobin. The ICU stay model incorporated all of the above terms with the exception of age and sex, which were excluded, and with the addition of anaesthetist, which was found to be significant in predicting ICU stay. This analysis showed that, after adjustment for these covariates, the administration of tranexamic acid had an independent effect on administration of red blood cells, other blood products and return to theatre for bleeding. The odds of receiving a transfusion of red blood cells, fresh frozen plasma or any blood product overall, or returning to theatre, were lower in patients who received tranexamic acid (Table 7). The level of significance was higher than in the univariate analysis. The administration of tranexamic acid did not affect the length of stay in ICU in this analysis.

**Table 7:** Multivariate analysis of the effect of administration of Tranexamic acid on the outcomes of interest.

Risk	Odds Ratio (95% CI)	p value
RBC transfusion	0.58 (0.47 – 0.71)	< 0.001
FFP transfusion	0.57 (0.43 – 0.75)	< 0.001
Any blood product transfusion	0.58 (0.48 – 0.71)	<0.001
Return to theatre	0.44 (0.32 – 0.60)	<0.001
ICU stay	0.83 (0.67 – 1.04)	0.100

Multivariate analysis of the effect of administration of Tranexamic acid on the outcomes of interest. The multivariate analysis showed that the odds of receiving a transfusion of any type of product, or returning to theatre for bleeding, were less in patients in tranexamic acid group. (RBC – red blood cells, FFP – fresh frozen plasma, ICU – intensive care unit, CI – confidence interval) (* = p value < 0.05, statistically significant)

Our subgroup analysis of patients transfused with red blood cells showed no difference in the number of units of red blood cells, fresh frozen plasma or all blood products transfused between the two groups (Table 8). There was no difference in the proportion of patients receiving a transfusion or staying more than 1 day in ICU between the groups, on excluding the patients who returned to theatre for re-exploration (Table 9).

**Table 8:** Subgroup analysis of patients who received a transfusion of red blood cells.

	Number of units (median, IQR)		
		
Type of transfusion	TA group (n = 1543)	NTA Group (n = 427)	p value
RBC	2 (1,4)	3 (1,5)	0.228
FFP	0 (0,0)	0 (0,0)	0.117
Total blood products	3 (2,6)	3 (2,6)	0.289

Subgroup analysis of patients who received a transfusion of red blood cells. In patients (n = 1970) who received a transfusion, the administration of tranexamic acid did not affect the number of units transfused. (p value – not significant, Mann-Whitney U test)

(TA – tranexamic acid, NTA – no tranexamic acid, RBC – red blood cells, FFP – fresh frozen plasma, IQR – inter quartile range)

**Table 9:** Sub group analysis, excluding patients returned to theatre for re-exploration of bleeding.

	Number of patients (%)		
		
Outcome measure	TA group (n = 3199)	NTA group (n = 763)	p value
RBC transfusion	1354 (42)	351 (46)	0.065
FFP Transfusion	187 (5.9)	52 (6.8)	0.312
Any blood product transfusion	1384 (43)	358 (47)	0.067
ICU stay > 1 day	627 (20)	161 (21)	0.351

Sub group analysis, excluding patients returned to theatre for re-exploration of bleeding. After excluding patients who returned to theatre for bleeding, there was no difference between the TA and NTA groups for the proportion of patients who received a transfusion or stayed in the intensive care unit for more than a day. (n = 3962, p value – not significant, Mann-Whitney U test) (TA – tranexamic acid, NTA – no tranexamic acid, RBC – red blood cells, FFP – fresh frozen plasma, ICU – intensive care unit)

The analysis of blood loss showed that the median (IQR) total blood loss was 575 mL (375, 875) in the NTA group compared to 450 mL (325, 675) in the TA group, a highly significant difference (p < 0.0001 Mann-Whitney test). Corresponding figures for 12 hours blood loss are 450 mL (300, 675) in the NTA group compared to 350 mL (250, 500) in the TA group (p < 0.001). Total blood loss for patients returned to theatre was 1959 mL (1444, 2925) for those patients receiving tranexamic acid compared with 2000 mL (1550, 2675) for those not receiving tranexamic acid (a non significant difference). In contrast, a highly significant difference (p < 0.001) in total blood loss was observed in those patients not returned to theatre when comparing the ones who had received tranexamic acid (TA group: 425 mL (325, 625)) compared with the ones who did not (non TA group: 550 mL (375, 775).

An univariate analysis of these data showed that a reduction in blood loss of 21% was expected in the TA compared to the NTA group. This analysis also indicates with high statistical significance (all p < 0.001) that postoperative blood loss is greater in males, those undergoing non-elective surgery, those undergoing CABG or CABG and valve surgery (compared to just valve surgery), those receiving aspirin or clopidogrel, older patients, patients with a high BMI, high Euroscore or long bypass time. The subsequent multivariate modelling indicated that a reduction in blood loss of 24% is expected in the TA compared to the NTA group.

## Discussion

In our hospital, the administration of tranexamic acid is an independent factor impacting on the exposure to red blood cells, fresh frozen plasma or any blood product. It is also an independent factor impacting on the return to theatre for re-exploration. These findings, obtained from the prospective observation of a very large number of patients in our routine clinical practice, support the translation of the evidence gathered through randomised clinical trials [1-3]. The administration of tranexamic acid indeed significantly reduced the amount of blood loss. Interestingly, this difference was identified for the whole population and the patients who did not return for re-exploration, but not significant for those patients who were taken back to theatre.

Two meta-analyses on the different pharmacological strategies used to decrease excessive blood loss in cardiac surgery have shown that lysine analogues decrease postoperative transfusion. The first showed that tranexamic acid compared to placebo reduced the proportion of patients receiving allogeneic blood transfusions (OR 0.50, 95% CI: 0.34–0.76, 12 trials, 882 patients) but without any significant effect on re-exploration for bleeding (OR 0.91, 95% CI: 0.37 – 2.23) [2]. The authors reported that the dosing of tranexamic acid and the blood transfusion guidelines were not uniform and the sample sizes of most of the studies included in the meta-analysis were small. They recommended conducting large randomised controlled trials with well defined transfusion guidelines to establish the best use of drugs used to minimise blood loss. In the second meta-analysis, Levi and colleagues showed that lysine analogues (tranexamic acid and epsilon aminocaproic acid) decreased perioperative blood loss, need for transfusion, need for re-exploration and mortality [3]. The likelihood of receiving a transfusion was found to be lower for patients treated with lysine analogues compared to those treated with placebo (OR 0.46, 95 % CI: 0.34–0.64, 14 studies, 801 patients). The need for re-thoracotomy for any reason within 72 hours of the operation was also decreased in patients treated with lysine analogues (OR 0.44, 95% CI: 0.22–0.99, 11 studies, 1026 patients). Similar trends were observed when only trials with complicated cardiac surgery were analysed. However, these studies did not report the effects of these drugs in uncomplicated cardiac surgery alone and did not differentiate the effects of tranexamic acid and epsilon aminocaproic acid separately.

This evidence was supported in a systematic review on use of antifibrinolytics in minimising the perioperative blood transfusion after any major surgery [1]. The authors reported that tranexamic acid reduced the rate of red blood cell transfusion by 34% (RR 0.66, 95% CI: 0.54 – 0.81, 18 trials, 1342 patients). In those receiving transfusion, tranexamic acid resulted in a saving of 1.03 units of RBC (95% CI: 0.67 to 1.39). However, the authors highlighted the significant heterogeneity among the trials, some evidence of publication bias and the paucity of collected data.

The common problems highlighted by these various reviewers are the heterogeneity of the study population, heterogeneity of the methods used in the trials (dosing regimen, monitoring of bleeding and coagulation and transfusion trigger) and the small number of patients studied. Though all these reviews showed that tranexamic acid reduced the proportion of patients needing a transfusion, they did not exclusively study the effects of routine use of tranexamic acid for all primary cardiac operations. Further, the results from such meta-analyses must be interpreted with caution as it has been shown in the past that larger, randomised, double blinded studies following meta-analysis have produced contradictory results [21,22]. For the same reasons, the authors of these reviews recommended conducting large randomised, double blinded, placebo controlled studies. However, no large trial with adequate power to identify the effects of the tranexamic acid administration compared to placebo on transfusion is available.

A few trials, with relatively large numbers of patients, comparing the effects of tranexamic acid with either placebo or other antifibrinolytic agents have indeed been conducted since. In one study, Armellin et al compared the effects of high dose of tranexamic acid with placebo in 300 patients undergoing aortic valve replacement [9]. They showed reduction in postoperative bleeding, usage of red blood cells and fresh frozen plasma and the proportion of patients receiving blood products. In another study of primary elective cardiac operations, Casati et al compared the effects of tranexamic acid and high dose aprotinin in 1040 patients [10] and found that tranexamic acid and aprotinin had similar effects on postoperative bleeding and allogeneic transfusion. They recommended tranexamic acid as a cheaper alternative in primary cardiac operations. Similarly, Casati et al studied the effects of aprotinin, tranexamic acid and epsilon aminocaproic acid in 210 elective cardiac surgical patients and showed that the combined costs of the pharmacological and transfusion treatment were significantly lower in the tranexamic acid group [11]. Diprose et al compared the effects of tranexamic acid with aprotinin and placebo in 180 patients who underwent any cardiac operation [12] and showed aprotinin reduced bleeding and transfusion significantly, when compared to tranexamic acid or placebo. However, the combined drug and transfusion costs were the least in the tranexamic acid group. Hekmat et al compared the effects of aprotinin and tranexamic acid in 120 patients and showed that tranexamic acid can be a cost-effective alternative in primary CABG patients [13]. Nuttall et al compared the effects of tranexamic acid with or with out intraoperative autologous blood collection (combined therapy), aprotinin and placebo in 160 patients [14]. They showed tranexamic acid, in combination with autologous blood collection, provided a similar reduction in bleeding and transfusion when compared to aprotinin and was less expensive. All these studies have repeatedly shown that tranexamic acid provides a cheap means for reducing transfusions in routine cardiac operations.

Intriguingly, those studies have all varied in the selected dose of the tranexamic acid to be administered and none established which dose is the most efficacious. Some dosages have been studied, such as 10 mg.kg^-1 ^followed by an infusion of 1 mg.kg^-1^.h^-1 ^[23-25], or 20 mg.kg^-1 ^and 100 mg.kg^-1 ^as an intravenous bolus [26,27]. These recommendations are based on small studies and further, larger, studies to determine the effective dose of tranexamic acid are lacking.

It appears however that a randomised control trial to convince those that remain sceptical of the effect of tranexamic acid is not ethical. Using the available information instead of a randomised, controlled trial to identify a clinical effect or to solve a clinical problem has been described in the literature and may be a more frequent occurrence with the development of the automatic collection of a vast quantity of data at the bedside [28]. This method can be useful if the data collected are reliable and appropriate statistical tests are used for analysis [29-31]. Moreover, a multitude of risk factors are associated with postoperative bleeding following cardiac surgery [32] and how (or if) they have been controlled for in the randomised control trials is unclear, and groups may ultimately not be matched. A randomised controlled trial accounting for those, and mimicking clinical reality, would be very expensive and would divert a large number of patients from other trials, designed to answer other important clinical questions. In this study, we have used the data collected prospectively to identify the effects of tranexamic acid administration in our routine patient population. Multivariate logistic regression was used to minimise the bias by adjusting for other confounding factors. This study unifies, in a large group of patients, the evidence from randomised controlled trials showing that tranexamic acid administration is an independent factor impacting on the exposure to allogeneic red blood cells, fresh frozen plasma or any blood product and on the return to theatre for re-exploration in patients who underwent a primary cardiac operation.

Interestingly, tranexamic acid administration did not impact on the length of intensive care unit (ICU) stay though it significantly decreased the incidence of re-exploration. This is different to findings from randomised studies but can be easily explained by the fact that most patients only stayed in ICU for one night and data were regarded as binary (stays 1 night or more). Discharge criteria, in real life, are dependent on time of day when the patient was operated, intensive care bed requirements and the availability of ward beds.

Though the proportion of patients receiving a transfusion was lower in the tranexamic acid group, our subgroup analysis showed that among the transfused patients, there was no difference in the number of units transfused between the groups. As the data is highly positively skewed, further analysis and interpretation of these results is difficult. On exclusion of patients who returned to theatre for re-exploration, the proportion of patients who received a transfusion or who stayed in ICU for more than one night were less in tranexamic acid, without reaching statistical significance. The proportion of patients who returned to theatre was lower in the tranexamic acid group, as previously described. As these patients were at a high risk of receiving a transfusion, exclusion of these patients possibly removed the effect of tranexamic acid administration on the outcome measures, though the proportion of patients who returned to theatre was much less compared to the proportion of patients who received a transfusion. A legitimate criticism of this paper could be that patients returned to theatre for an obvious surgical reason should have been excluded from the analysis. We elected not to do so as to record accurately and without bias the cause of return is difficult and likely to be inaccurate due to the inherent bias of the operator in the absence of a neutral adjudicator. It may also be that patients with more intra-operative blood loss are more likely to return later as the surgeon will have had greater difficulties in ensuring correct haemostasis.

While this observational study lacks characteristics inherent to a well conducted randomised, double blinded, placebo controlled trial, it adds to the wealth of knowledge already available by studying the effect of a therapy in a large group of patients subjected to a normal clinical pathway. We used multivariate logistic regression in order to minimise a selection bias on treatment allocation. Our large sample size means that the statistical tests have good power. However, for the same reason, these tests can detect very small differences as statistically significant, such as showing that patients receiving tranexamic acid were older, had a higher EuroSCORE and had longer bypass times, but these differences were not clinically significant. Other statistically significant differences were observed in relation to postoperative blood loss, but some of the highly significant differences were small due to the large sample size (i.e. the median blood loss for patients receiving clopidogrel was 500 mL compared with 450 mL for those patients not receiving clopidogrel). In the absence of randomisation, a subjective effect cannot entirely be discarded by which physicians may be unconsciously biased towards administering less transfusion to those patients having received tranexamic acid. However, we believe that this is unlikely to have tainted our primary end-points as (1) adherence to the institutional protocol was similar for both patients receiving or not tranexamic acid (In the TA group 1207 (78%) of transfused patients were transfused according to guidelines compared with 319 (75%) in the NTA group, the difference is non-significant (p = 0.124, Chi-squared test)); (2) a significant difference in the amount of blood loss measured in those patients treated with tranexamic acid when compared with those who were not; (3) the fact that the size of the institution and clinical pathways usually prevent clinicians prescribing postoperative transfusion and involved in the decision to take back to be aware if the administration of tranexamic acid effectively took place (as all patients should have received it).

We did not investigate the mortality and morbidity following the use of tranexamic acid, neither graft patency nor the incidence of deep vein thrombosis or pulmonary embolism as data are not available in that regard.

This study does not suggest that tranexamic acid is a safer alternative to other antifibribolytic, neither does it address the possibility that it may affect morbidity as recently reported with aprotinin [15,16,18-20].

## Conclusion

In conclusion, based on the analysis of data obtained for 4191 patients operated in our institution, a standard dose regimen of tranexamic acid reduces the need for transfusion of any blood product in patients undergoing primary cardiac surgery.

## Competing interests

Dr Vuylsteke is receiving consultancy fees from by Novo Nordisk, in relation to the development of Novoseven^®^.

No other competing interests.

## Authors' contributions

AV conceived the study and participated in its design and helped to draft the manuscript. PS participated in the design of the study and drafted the manuscript. CG collected all data used in the study and helped to draft the manuscript. FC performed the statistical analyses.

All authors read and approved the final manuscript.

## Pre-publication history

The pre-publication history for this paper can be accessed here:


